# Activation of Langerhans-Type Dendritic Cells Alters Human Cytomegalovirus Infection and Reactivation in a Stimulus-Dependent Manner

**DOI:** 10.3389/fmicb.2016.01445

**Published:** 2016-09-14

**Authors:** Roxanne Coronel, Desyree M. Jesus, Lucia Dalle Ore, Joe S. Mymryk, Laura Hertel

**Affiliations:** ^1^Center for Immunobiology and Vaccine Development, Children's Hospital Oakland Research InstituteOakland, CA, USA; ^2^Department of Microbiology and Immunology and Department of Oncology, The University of Western OntarioLondon, ON, Canada

**Keywords:** langerhans cells, dendritic cells, cytomegalovirus, host defense, infection restrictions

## Abstract

Oral mucosal Langerhans cells (LC) are likely to play important roles in host defense against infection by human cytomegalovirus (CMV). We previously showed that *in vitro*-differentiated immature LC (iLC) populations contain smaller amounts of infected cells but produce higher yields than mature LC (mLC) cultures, obtained by iLC stimulation with fetal bovine serum (FBS), CD40 ligand (CD40L) and lipopolysaccharide (LPS). Here, we sought to determine if exposure to select stimuli can improve LC permissiveness to infection, if specific components of the mLC cocktail are responsible for lowering viral yields, if this is due to defects in progeny production or release, and if these restrictions are also effective against reactivated virus. None of the stimuli tested extended the proportion of infected cells to 100%, suggesting that the block to infection onset cannot be fully removed. While CD40L and FBS exerted positive effects on viral progeny production per cell, stimulation with LPS alone or in combination with CD40L was detrimental. Reductions in viral titers were not due to defects in progeny release, and the permissive or restrictive intracellular environment established upon exposure to each stimulus appeared to act in a somewhat similar way toward lytic and latent infections.

## Introduction

Langerhans-type dendritic cells (LC) are the only type of innate immune cells found in the epithelial layer of human oral mucosae, where they provide an initial line of defense against invading microorganisms (Barrett et al., 1996; Allam et al., 2003, 2006, 2008; Cutler and Jotwani, 2006; Novak et al., 2008). Acquisition of human cytomegalovirus (CMV), a source of serious disease in newborns, transplant recipients, and AIDS patients, normally occurs early in life by contact between contaminated bodily fluids, such as urine and saliva, and host oronasal mucosae. Limited replication within mucosal cells at the time of infection is followed by viral spread to the bone marrow, where latency is established (Pass, 2001; Britt, 2007; Mocarski et al., 2007). Intermittent bouts of reactivation then release virus back in the urine and saliva, thus promoting viral spread to new hosts.

Being present at densities of 20–80 cells/mm^2^ in the outermost layers of the oral mucosa (Cruchley et al., 1989, 1994; Lasisi et al., 2013; Upadhyay et al., 2013; Omine et al., 2015), LC are among the first cell types to encounter CMV during entry, together with epithelial cells. Their differentiation from CD34^+^ hematopoietic progenitors (Iijima et al., 2007), a well-known site of CMV latency (Minton et al., 1994; Mendelson et al., 1996; Zhuravskaya et al., 1997; Slobedman and Mocarski, 1999; Khaiboullina et al., 2004), was also shown to be associated with viral genome maintenance (Reeves et al., 2005a,b; Reeves and Sinclair, 2010), and reactivation (Reeves et al., 2005a,b; Reeves and Sinclair, 2010; Huang et al., 2012).

Although mucosal LC are conventionally considered immature, their actual maturation status is likely to be more heterogeneous, as the oral mucosa is normally colonized by a variety of microorganisms whose identity and pathogenicity depend on the health and hygiene of the host. Local contacts with infiltrating CD4^+^ and CD8^+^ lymphocytes expressing the CD40 ligand (CD40L), with bacterial products such as lipopolysaccharide (LPS) or with pro-inflammatory cytokines such as interleukin-1β (IL-1β), tumor necrosis factor α (TNF-α), and prostaglandin E2 (PGE2) can indeed stimulate mucosal LC maturation and migration (Yavuzyilmaz et al., 1995; Heasman et al., 1998; Orima et al., 1999; Loro et al., 2001; Orozco et al., 2006).

We previously showed that infection initiates in only a small fraction of *in vitro*-differentiated immature LC (iLC), as compared to mature LC (mLC) obtained by iLC stimulation with granulocyte-macrophage colony-stimulating factor (GM-CSF), fetal bovine serum (FBS), LPS and CD40L (Lauron et al., 2014; Coronel et al., 2015). Despite this and quite surprisingly, iLC produced higher amounts of viral progeny than mLC (Coronel et al., 2015), suggesting that CMV replication in these cells undergoes at least two blocks: one acting early and restricting infection onset to only specific cells within the population, and one acting later, reducing viral yields in mLC. Exposure of iLC differentiated from latently infected progenitors to the mLC cocktail also failed to enhance viral DNA replication and progeny production, suggesting that signaling by FBS, CD40L, and LPS in combination does not trigger robust CMV reactivation (Coronel et al., 2015).

In this work, we sought to determine: (1) if select stimuli can increase the proportion of LC that initiate infection by relieving the initial block; (2) if these stimuli also increase viral yields; (3) if the magnitude of progeny production can be predicted from the number of viral antigen^+^ cells detected at infection onset; (4) if populations containing large amounts of infected cells are also the most efficient at producing progeny; (5) if mLC's low yields are due to restrictions in progeny production or release; (6) which specific component(s) of the mLC cocktail is responsible for increasing the proportion of infected cells and for reducing viral yields, and (7) if similar restrictions also apply to progeny produced following viral reactivation from latency.

We show that iLC stimulation with the mLC cocktail, with pro-inflammatory cytokines or with CD40L, but not with FBS or LPS, increases the proportion of infected cells. Populations exposed to pro-inflammatory cytokines or CD40L also produced yields similar to those of iLC, while treatment with FBS, LPS, or the mLC cocktail reduced progeny amounts to different extents. A linear correlation was observed between the number of antigen^+^ cells present at infection onset and the amount of progeny produced at peak times, with the notable exception of mLC cultures. As the proportion of infected cells changed over time with different kinetics in each population, an inverse correlation was detected between the amount of progeny produced per cell and the total number of antigen^+^ cells present at late times post-infection, suggesting that conditions which support prolonged viral protein expression may reduce the ability of each infected cell to produce virus.

Populations matured using the mLC cocktail remained the only ones to display large(r) amounts of infected cells but extremely low yields, a phenotype possibly ascribable to the opposing effects of exposure to CD40L, which increased the proportion of infected cells and viral yields, and to LPS, which reduced both parameters. All LC populations contained approximately twice as much cell-associated than cell-free virus, suggesting that defects in progeny release are unlikely to be the source of mLC's low yields. By contrast, the number of cell-associated particles per infected cell ranged from ~0.2 in mLC to ~6 in FBS cultures, implying the existence of a severe block to progeny production in mLC populations. With the notable exception of pro-inflammatory cytokines, none of the tested stimuli increased the extent of viral reactivation beyond that observed in iLC cultures, indicating that the intracellular environments that restrict lytic viral replication also constrain viral reactivation. Together, these data highlight the remarkable functional plasticity of LC: instead of being strongly polarized toward either a fully permissive or a fully resistant phenotype, LC responses to CMV infection and reactivation follow a continuum whereby the strength and extent of the restrictions imposed vary depending on the nature and combination of the stimuli received.

## Materials and methods

### Cells and virus

Immature LC populations were derived from cord blood CD34^+^ hematopoietic progenitor cells (STEMCELL Technologies Inc., Vancouver, Canada, six different donors) as previously described (Lauron et al., 2014; Coronel et al., 2015). LC activation was induced by exposure to 1500 IU/ml of GM-CSF and 10% FBS (US origin, Gibco, Life Technologies, South San Francisco, CA), or 1500 IU/ml of GM-CSF and 200 ng/ml of CD40L (Peprotech, Rocky Hill, NJ), or 1500 IU/ml of GM-CSF and 250 ng/ml of LPS (Sigma-Aldrich, St. Louis, MO), or 1500 IU/ml of GM-CSF, 200 ng/ml of CD40L, and 250 ng/ml of LPS, or 1500 IU/ml of GM-CSF, 10% FBS, 200 ng/ml of CD40L and 250 ng/ml of LPS, or 125 U/ml of TNF-α, 400 IU/ml of IL-1β, 1000 IU/ml of IL-6 and 5 nmol/ml of PGE2 (all from BioVision, Inc., Milpitas, CA), or 1500 IU/ml of GM-CSF, 10% FBS, 200 ng/ml of CD40L, 250 ng/ml of LPS, 125 U/ml of TNF-α, 400 IU/ml of IL-1β, 1000 IU/ml of IL-6, and 5 nmol/ml of PGE2. CMV strain TB40/E was propagated on human foreskin fibroblasts (HFF) as previously described (Hertel et al., 2003).

### Cell infection

Immature LC harvested at day 8 post-differentiation were counted and re-plated in iLC or in each activation medium for 2 days prior to exposure to TB40/E virions at a calculated multiplicity of infection (MOI) of 10 for 4 h. Cells were then washed twice and re-plated in iLC or in each activation medium for 10 days. For reactivation studies, CD34^+^ cells were thawed and immediately exposed to TB40/E virions at a calculated MOI of 10 for 4 h, prior to washing and plating in iLC medium. At day 8, iLC were harvested, counted, and re-plated in iLC or in each activation medium for 10 days. Latently-infected LC harvested at day 2, 4, and 8 were counted and added at a density of 10^4^–10^5^ per 5 × 10^5^ confluent HFF in single wells of six well-plates. At day 8 of co-culture, plates were stained with Giemsa and plaques were counted.

### Immunofluorescence staining analyses

Cytospin preparations were fixed in 1% formaldehyde for 30 min at room temperature (RT), permeabilized in 0.5% Triton-X 100 for 20 min on ice, blocked with 40% FBS/40% goat serum for 30 min at RT and stained for the viral immediate-early (IE) proteins 1 and 2 (MAb810, 1:600, Light Diagnostics, Temecula, CA). Nuclei were labeled with Hoechst 33342 (0.2 mg/ml; Molecular Probes, Eugene, OR). Samples were viewed using a Nikon Eclipse E600 fluorescence microscope equipped with an iVision-Mac imaging software.

### Virus titrations

Titers of culture supernatants and cell-associated virus, obtained by sonication of LC pellets for ~1 s with a Branson Ultrasonics Sonifier 150, were determined by staining HFF with MAb810 at 24 h post-infection.

### Statistical analysis

Linear regression analyses of data distributions were performed using Prism 7.0. The cumulative distributions of any two data sets were compared using the Kolmogorov-Smirnov test. Differences were considered significant at *P* < 0.05.

## Results

### LC activation increases the number of infected cells but not viral yields

**Figure 1:**
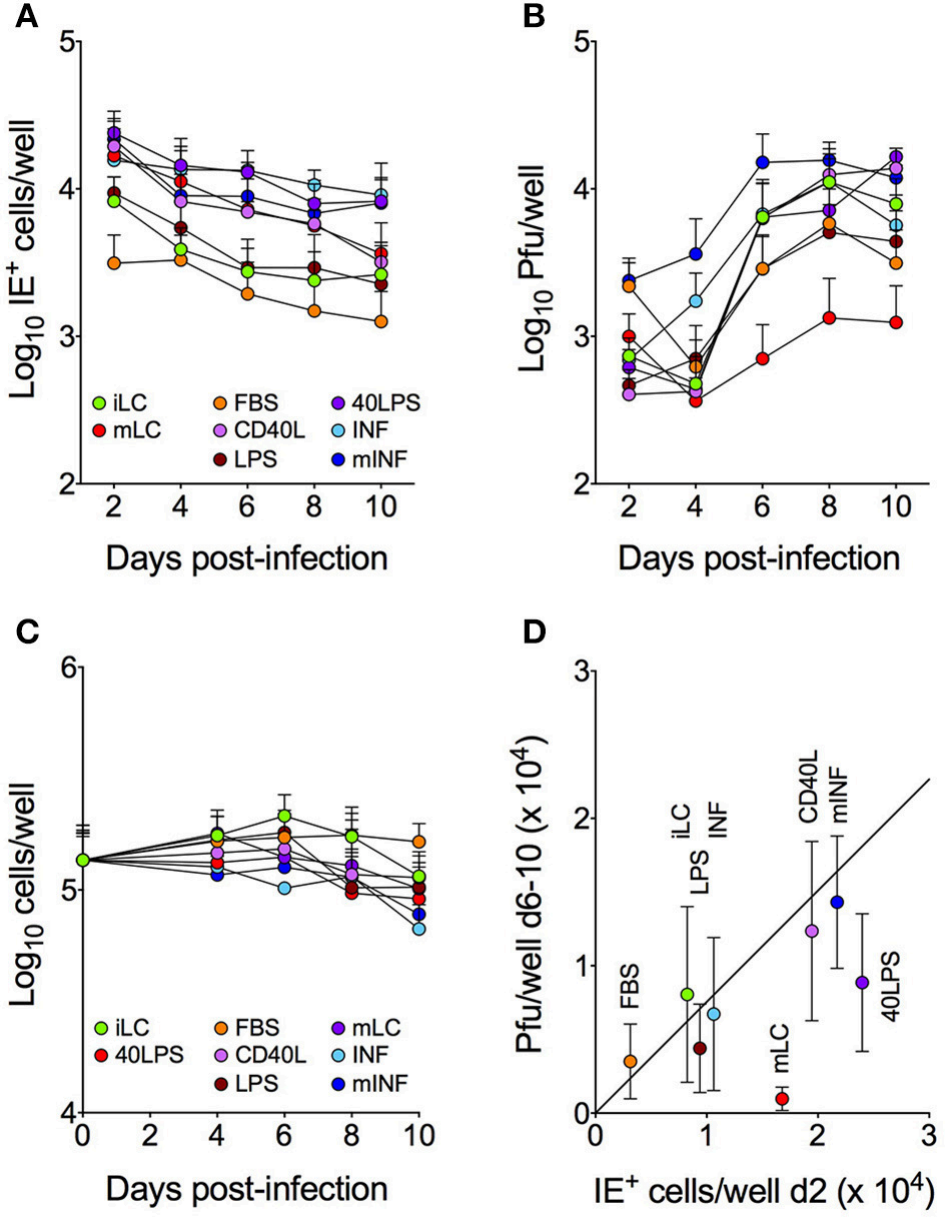
**Activation differentially affects the number of IE^+^ cells and progeny yields**. LC cultured under eight different conditions were infected with TB40/E at an MOI of 10, and subsequently maintained in each medium for 10 days, with cell and supernatant collections every 2 days. The total number of IE^+^ cells present in each well was determined by immunofluorescence staining analyses **(A)**, the amount of virus present in each supernatant was assessed by titration **(B)** and the total number of cells per well was counted **(C)**. The median number of cells expressing the IE proteins at day 2 was then plotted against the median number of plaque forming units (pfu) present in the supernatants collected from each well at days 6–10 **(D)**, and the best-fitting line modeling the relationship between the *X* and *Y* values of all data points except those of mLC cultures was identified by linear regression (*Y* = 0.7*X, R*^2^ = 0.87). Symbols represent the median and median absolute deviation values of data collected with LC derived from six different CD34^+^ cell donors in eight independent experiments, from three donors in two independent experiments **(C)** and from three donors in two independent experiments (40LPS condition).

Immature LC differentiated from the CD34^+^ progenitor cells of different donors were cultured for 2 days in one of seven activation media, consisting of GM-CSF/FBS, GM-CSF/CD40L, GM-CSF/LPS, GM-CSF/CD40L/LPS (40LPS), GM-CSF/FBS/CD40L/LPS (mLC medium; Lauron et al., 2014; Coronel et al., 2015), interleukin 6 (IL-6), IL-1β, PGE2, and TNF-α (pro-inflammatory mix, INF) (Jonuleit et al., 1997; Beck et al., 2003; Sénéchal et al., 2004), or GM-CSF, FBS, CD40L, LPS, IL-1β, IL-6, PGE2, and TNF-α (mLC and pro-inflammatory mix combination, mINF). Cells were then infected with CMV strain TB40/E at a calculated MOI of 10, and the proportion of cells expressing the viral immediate-early (IE) proteins 1 and 2 at day 2, 4, 6, 8, and 10 was determined. While culture in CD40L, 40LPS, mLC, INF, and mINF conditions increased the total number of infected cells by ~3-fold at early times as compared to iLC, exposure to FBS or LPS had no boosting effects (Figure 1A). The CD40L, 40LPS, mLC, INF, and mINF data distributions were also significantly different from that of iLC according to two sample, unpaired Kolmogorov-Smirnov (KS) tests (*P*-value for each comparison <0.0001), while those of FBS and LPS were not. Importantly, none of these conditions increased the proportion of infected cells to 100%, as observed in high MOI infections of fully susceptible cells. This suggests that only a specific subset of LC is permissive to CMV infection, and that this subset can only be slightly expanded by cell activation.

Consistent with our previous findings using CMV strain TB40-BAC4 and AD169*var*ATCC (Coronel et al., 2015), infection of iLC with TB40/E was productive (Figure 1B). Progeny was also made by activated cells, with CD40L, 40LPS, INF, and mINF cultures producing similar amounts of virus as iLC at peak times, FBS and LPS populations producing ~2-fold less, and mLC ~10-fold less. The kinetics of viral replication in INF and mINF treated cells were also slightly different from the rest, as yields started to rise from day 4 instead of from day 6 (Figure 1B). Only the mLC data distribution, however, was significantly different from that of iLC according to KS tests (*P* < 0.0007). Finally and importantly, these differences were not due to extensive cell death affecting some conditions (e.g., mLC) more than others, as the number of cells per well did not display dramatic variations among cultures (Figure 1C).

### The number of infected cells present at infection onset is predictive of the magnitude of progeny production

The number of CMV particles produced at peak times upon high MOI infection of permissive cells is usually directly proportional to the initial amount of infected cells (Coronel et al., 2015). To assess if this was also true for LC cultures, the median number of particles per well present at day 6–10 was plotted against the median number of IE^+^ cells detected at day 2 (Figure 1D). A linear relationship was observed between these two parameters for most conditions (*Y* = 0.7*X, R*^2^ = 0.87), whereby for each new IE^+^ cell gained at day 2, the median number of particles per well is increased by 0.7 units. Importantly, data from 40LPS and mLC cultures did not fit with this equation, displaying yields per cell 2-fold (40LPS) and 11-fold (mLC) lower than expected (Figure 1D).

### Populations containing large amounts of infected cells are not the most efficient at producing progeny

**Figure 2:**
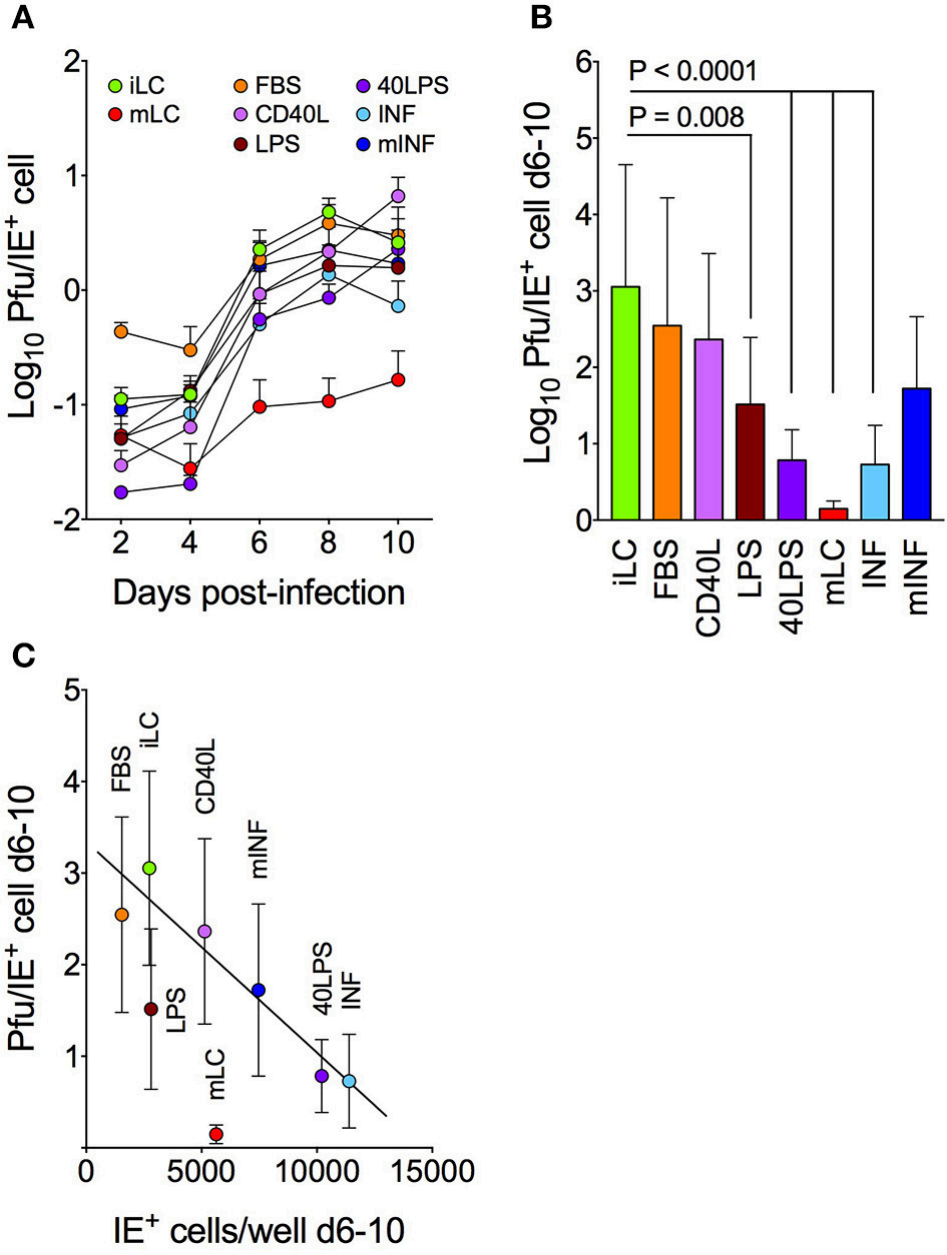
**Progeny production efficiencies inversely correlate with the number of infected cells present at late times post-infection**. Immature and activated LC were infected with TB40/E at an MOI of 10, and the amount of progeny per IE^+^ cell released in the supernatant during the entire time course **(A)** and at peak times (days 6–10, **B**) was calculated. The median number of pfu/IE^+^ cell was then plotted against the median number of cells expressing the IE proteins in each well at the same time **(C)**. The best-fitting line modeling the relationship between the *X* and *Y* values of all data points except those of mLC and LPS cultures was identified by linear regression (*Y* = –0.0002*X* + 3.3, *R*^2^ = 0.92). Symbols represent the median and median absolute deviation values of data collected with LC derived from six different CD34^+^ cell donors in eight independent experiments, and from three donors in two independent experiments (40LPS condition). The *P*-values for pairwise comparisons of data distributions using the KS test are shown.

Although the extent of IE protein expression was enhanced and prolonged in CD40L, 40LPS, INF, and mINF conditions (Figure 1A), yields failed to increase proportionally (Figure 1B). Consequently, none of the tested treatments improved the efficiency of progeny production of iLC cultures (Figure 2A). Rather, exposure to LPS, 40LPS, mLC, and INF significantly worsened it (Figure 2B).

Interestingly, an inverse correlation was also observed between the median number of IE^+^ cells present in each well at day 6–10 and the median number of particles produced by each IE^+^ during the same period of time, suggesting that the efficiency of progeny production decreases when the number of infected cells increases (Figure 2C). Data distributions could be modeled by the best fit linear equation *Y* = −0.0002*X* + 3.3 (*R*^2^ = 0.92), according to which acquisition of a new IE^+^ cell reduces the efficiency of progeny production by ~0.0002 pfu. Mature LC populations represented, once again, an exception to this rule, with yields ~13-fold lower than expected. Interestingly, LPS-treated cells also appeared to be less efficient than expected: despite containing roughly the same number of IE^+^ cells as iLC, these cultures produced about half as much virus. These data suggest that conditions which support prolonged IE protein expression do not concomitantly enhance the ability of infected cells to produce progeny, possibly due to the presence of one or more restrictions to viral genome replication or to virion assembly, maturation, and release.

### Loss of progeny production efficiency is not due to defects in particle release

**Figure 3:**
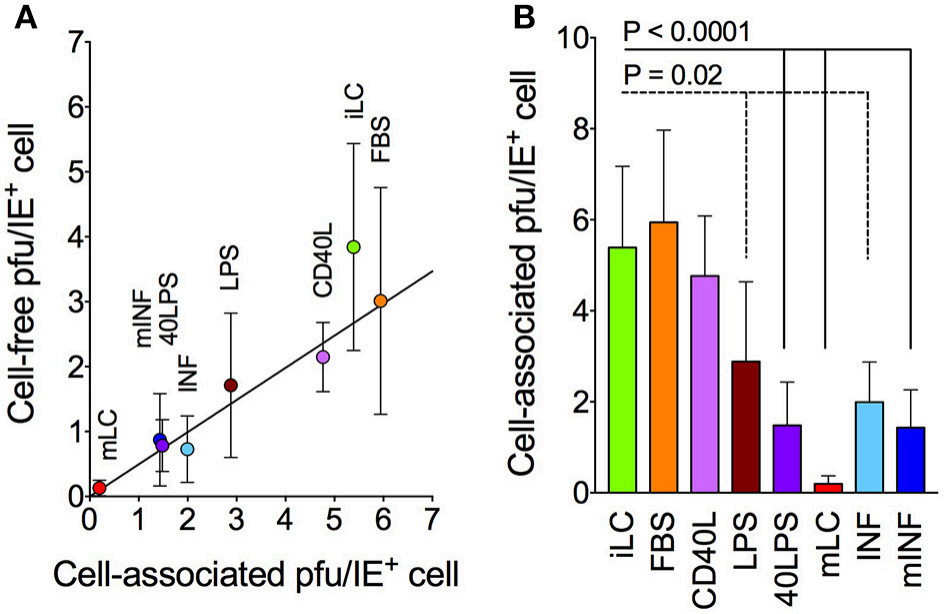
**Cell-free and cell-associated progeny yields in infected LC cultures**. Immature and activated LC were infected with TB40/E at an MOI of 10, and the amount of cell-associated virus per IE^+^ cell present at days 6–10 was calculated **(B)** and plotted against the amount of cell-free virus per IE^+^ cell present in each medium during the same time frame **(A)**. The best-fitting line modeling the relationship between the *X* and *Y* values of all data points was identified by linear regression (*Y* = 0.5*X, R*^2^ = 0.96). Symbols represent the median and median absolute deviation values of data collected with LC derived from three different CD34^+^ cell donors in three independent experiments, and from three donors in two independent experiments (40LPS condition). The *P*-values for pairwise comparisons of data distributions using the KS test are shown.

We previously showed that the intracellular content of viral genomes from CMV strain AD169*var*ATCC and TB40-BAC4 progressively declined over time in iLC populations, reaching values 20–25-fold lower than input, while genome loss in mLC was slower and much less dramatic, halting at values 4–5-fold lower than input (Coronel et al., 2015). In addition, the distribution of viral genomes per cell in infected mLC populations was consistent with the occurrence of viral DNA replication (Coronel et al., 2015). We thus speculated that the low efficiency of progeny production of mLC populations could be due to defects in viral particle production or release, rather than to impairments in viral genome replication. To test this hypothesis, the amount of cell-associated virus per IE^+^ cell was plotted against the amount of cell-free virus per IE^+^ cell present in each culture (Figure 3A). Very interestingly, all LC populations appeared to release approximately half of the virus produced (*Y* = 0.5*X, R*^2^ = 0.96), suggesting that the low yields observed in mLC and in other cultures (Figure 2B) are unlikely to be due to defects in progeny release. Instead, progeny production *per se* may be impaired, as the mLC, LPS, 40LPS, INF, and mINF data distributions were significantly different and lower than that of iLC, FBS, and CD40L (Figure 3B). While this impairment may still be the result of reduced viral genome replication in LPS, 40LPS, INF, and mINF cultures, a block in particle maturation is more likely to be the cause in mLC populations.

### CD40L and LPS exert opposing effects on infection progress

Despite containing higher amounts of IE^+^ cells than iLC cultures (Figure 1A), mLC yields were dramatically lower (Figure 1B), likely because of defects in progeny production (Figure 3). While exposure to CD40L alone increased the number of infected cells but did not boost yields, exposure to LPS alone did not affect the number of IE^+^ cells but reduced progeny amounts relative to those of iLC. Consequently, the efficiency of progeny production per cell was high in CD40L cultures, but significantly lower in LPS-treated cells. The combination of CD40L and LPS led to additional increases in the number of IE^+^ cells but not in titers, causing yields per cell to drop (Figures 2A,B, 3B). This suggests that CD40L and LPS may exert opposing effects on infection progress, with CD40L stimulating IE protein expression and LPS inhibiting progeny production. Neither stimulus, however, alone or in combination, recapitulated the exact phenotype of mLC cultures, which appears to require the addition of FBS. This suggests that the negative effects of LPS, rather than the positive influence of CD40L, are enhanced by FBS, even though, when used in isolation, FBS yields per cell are similar to those of iLC and CD40L populations (Figures 2A,B, 3B). Interestingly, addition of the mLC cocktail to the IL-6, IL-1β, PGE2, and TNF-α mix in the mINF samples did not dramatically lower the efficiency of progeny production, suggesting that signaling by pro-inflammatory cytokines may partially relieve the block imposed by the combination of FBS, CD40L, and LPS.

### Viral reactivation is not enhanced by LC activation

In directly infected cells, viral replication initiates in the presence of the tegument proteins, released into the cell upon entry, and from DNA molecules newly introduced into the nucleus. Viral reactivation from latently infected cells, by contrast, initiates from genomes that have been maintained within the cell nucleus for prolonged periods of time, and in the absence of tegument proteins.

We thus wondered if the observed effects of cell activation on lytic replication would also apply to reactivation from latency. CD34^+^ cells from the same donors used in lytic infection assays were exposed to TB40/E virions at a calculated MOI of 10 for 4 h, prior to culture in iLC medium for 8 days. At the end of the differentiation period, a fraction of latently infected iLC was plated onto uninfected HFF, and the number of plaques that developed after 8 days of co-culture was counted. The remaining iLC were re-plated in either iLC conditions, or in each medium. Supernatants and cells were then collected at day 2, 4, and 8 (corresponding to day 10, 12, and 16 post CD34^+^ cells infection), and used in titration assays or in reactivation experiments, respectively.

Consistent with our previous results using CMV strain AD169*var*ATCC and TB40-BAC4 (Coronel et al., 2015), IE^+^ cells were not observed in any culture at any time (not shown), indicating that TB40/E infection of CD34^+^ cells was not lytic. Also in agreement with our previous findings, extremely low amounts of viral particles were detected in the supernatant of latently infected iLC and mLC at day 2, 4, and 8 after re-plating. Similar results were obtained in each condition (Table 1, Pfu), suggesting that differentiation of latently infected CD34^+^ cells into iLC, and their subsequent exposure to activation stimuli, is not sufficient to trigger robust viral reactivation. By contrast, ~10-fold higher numbers of plaques per cell were observed upon co-culture of each LC type with HFF (Table 1, Plaques), supporting our previous conclusion that additional stimulation beyond that provided by the activation cocktails, and possibly consisting of soluble factors secreted by HFF or LC during the co-culture period, is required to induce full reactivation (Coronel et al., 2015). Alternatively, contact with HFF may facilitate the release of reactivated virus from latently infected LC via enhanced cell-to-cell transmission.

**Table 1:** **Viral particle content per 10^5^ cells in the supernatant of cells differentiated from latently infected CD34^+^ progenitors (Pfu) and number of plaques per 10^5^ cells obtained after their co-culture with HFF (Plaques)**.

	**Donor 1**			**Donor 2**		
	**Day 2**	**Day 4**	**Day 8**	**Day 2**	**Day 4**	**Day 8**
iLC pfu	2	2	0	3	1	0
iLC plaques	10	50	10	80	30	10
FBS pfu	1	0	2	6	0	0.5
FBS plaques	40	30	10	10	40	10
CD40L pfu	0	0	0	1	0	0.7
CD40L plaques	10	50	0	10	50	10
LPS pfu	0	1	0	0	0	0
LPS plaques	10	0	0	0	0	10
mLC pfu	2	0	0	0	0	0
mLC plaques	30	10	0	10	0	0
INF pfu	2	0	1	0	2	0
INF plaques	10	60	50	30	50	50
mINF pfu	2	0	1	2	1	0
mINF plaques	10	40	30	30	30	20

**Figure 4:**
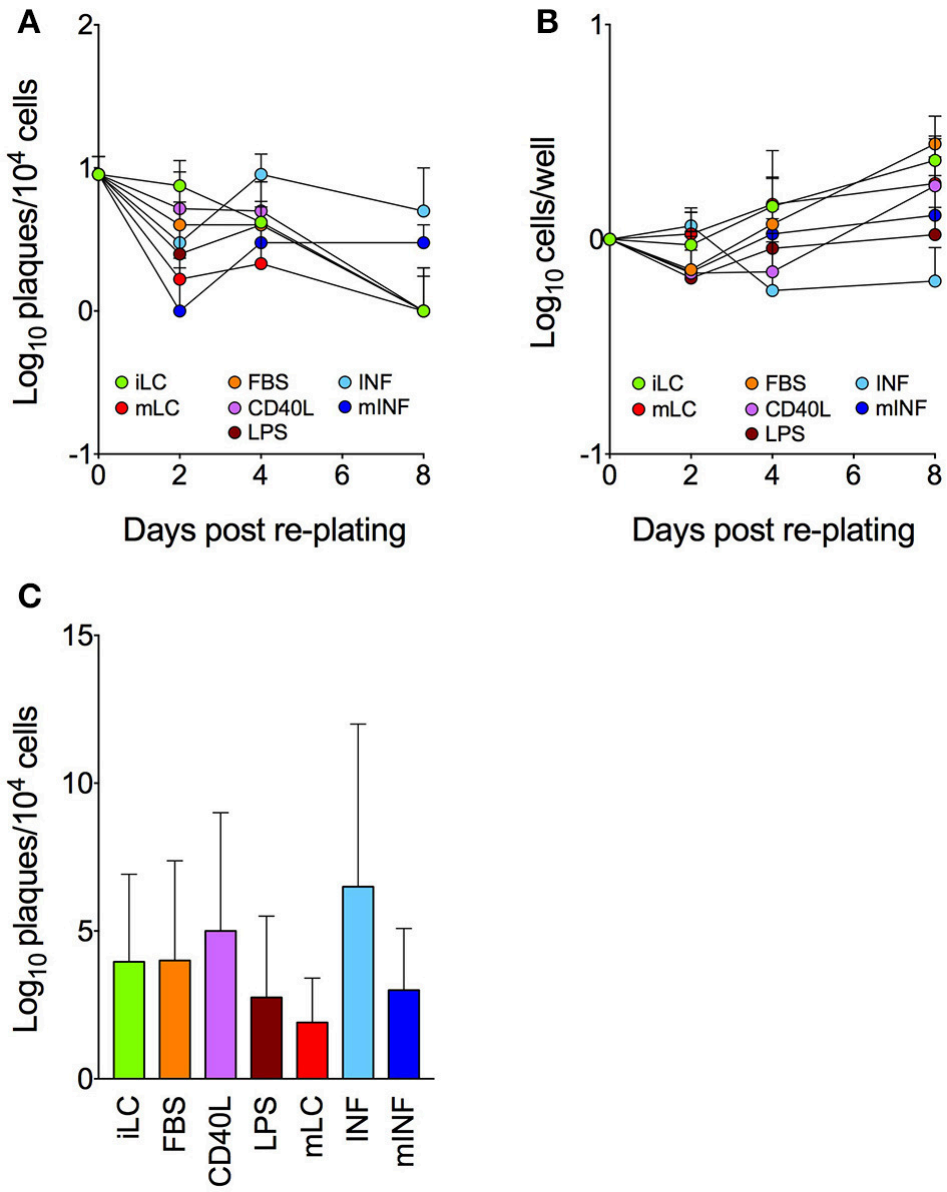
**Efficiency of viral reactivation in latently infected LC populations**. CD34^+^ cells were exposed to TB40/E virions at a calculated MOI of 10 for 4 h, washed and cultured in iLC differentiation medium for 8 days. A portion of latently infected iLC harvested at day 8 was then plated onto uninfected HFF, and the number of plaques that developed during a period of 8–10 days was counted (day 0). The remaining iLC were re-plated in either iLC conditions or in each activation medium. Cells were collected at day 2, 4, and 8, counted and co-cultured with uninfected HFF for 8–10 days. The number of plaques that developed in each condition and at each time post re-plating **(A)**, the total number of cells per well **(B)** and the overall median number of plaques **(C)** were calculated. Graphs show median and median absolute deviation values of data collected with latently infected LC derived from five different CD34^+^ cell donors in two independent experiments.

Plaques were consistently detected in co-culture wells seeded with iLC harvested at the end of the CD34^+^ cells differentiation period (Figure 4A). While the number of reactivation events produced by iLC populations progressively declined over time, a drop from day 0 to day 2 was observed in all other conditions. This was followed by a stabilization at day 4, and by another decrease to values ~10-fold lower than those of day 0 in iLC, FBS, CD40L, LPS, and mLC cultures. Importantly, this decline in reactivation frequencies was not due to widespread cell death, as cell numbers increased in all cultures except INF during the same time frame (Figure 4B). In sharp contrast, the number of plaques detected in INF and mINF cultures rose from day 2 to 4 and remained high at day 8, ending at values only ~2-fold lower than those of day 0. Intriguingly, the median number of plaques produced by mLC and LPS cultures was lower than that of iLC, FBS, and CD40L populations (Figure 4C), thus mirroring the differences observed in lytically infected cultures (Figures 2B, 3B), without however reaching the same degree of statistical significance. Thus, although the positive or negative influences exerted by each treatment on viral replication may be similarly effective during lytic and latent infection, their effects are likely altered or clouded by the presence of factors secreted during the co-culture period and/or ensuing LC contact with HFF.

## Discussion

Horizontal CMV transmission is thought to occur by contact between virions and host oronasal mucosae, whose outer layers are exclusively populated by epithelial cells and LC. These cell types are thus likely to play important roles in CMV acquisition and intra- as well as inter-host viral spread.

Both the phenotype and functions of mucosal LC can be strongly modulated by contact with soluble factors, other immune cells, and pathogenic or commensal microorganisms. LC responses to incoming CMV virions are thus likely to vary depending on their degree and mode of activation. In this work, we sought to assess the impact of different activation stimuli on the ability of *in vitro*-generated LC to support CMV infection and reactivation.

Consistent with our previous data (Hertel et al., 2003; Lauron et al., 2014; Coronel et al., 2015), we found that IE expression in iLC populations is very limited, and ~20-fold lower than what expected for high MOI infections of fully permissive cells such as HFF (Coronel et al., 2015). These findings provide further support to our hypothesis that a block restricting transcription or translation of the UL122/123 genes, encoding the IE1/IE2 proteins, exists in a large proportion of iLC cultures (Lauron et al., 2014). The fact that exposure to select activation stimuli increases the number of infected cells (Figure 1) suggests that some signaling pathways can partially relieve this block. We think that these signals are most likely to act directly on viral gene transcription, rather than on virion entry or intracellular trafficking, as iLC contain more viral particles than mLC at early times post-infection (Lauron et al., 2014), similar numbers of viral genomes reach the nucleus of both cell types (Lauron et al., 2014), and prolonged exposure to the inoculum does not substantially increase the proportion of IE^+^ cells (not shown). The antiviral activities of nuclear domain 10 bodies (ND10) are also unlikely to be the main source of this block, as expression of specific ND10 components such as PML and hDaxx was increased, rather than reduced, in cells exposed to IL-6 (Hubackova et al., 2012), IL-1β (Heuser et al., 1998; Ha et al., 2006), or CD40L (Salomoni et al., 2006). The enhancement of viral gene transcription ensuing signaling by activation stimuli may occur, for instance, if the abundance, availability or activation status of transcription factors such as the major immediate-early promoter activators NF-kB, CREB, and Sp1 (Hunninghake et al., 1989; Sambucetti et al., 1989; Lang et al., 1992) is altered. Alternatively, signaling may affect the degree of chromatinization of viral genomes, or change their epigenetic landscape, tilting the balance in favor of transcription activation by promoting histone acetylation and/or demethylation. Clearly, additional studies are needed to define the exact mechanisms acting in each population, keeping in mind that these are likely to differ, either qualitatively or quantitatively, in cultures exposed to each activation mix.

Similar to other permissive cells, the viral yields of each population (with the exception of mLC) were directly proportional to the amount of infected cells present at infection onset (Figure 1). The calculated number of particles produced by each initially infected cell (0.7 pfu/cell), however, remained substantially lower than that of other cell types (10 or more pfu/cell; Coronel et al., 2015), and could not be increased by LC activation. Also in contrast to other cell types, the efficiency of progeny production per cell was larger in populations containing smaller number of infected cells (Figure 2). This could be due to the fact that the initial pool of progeny-producing cells present in populations with low numbers of IE^+^ cells such as iLC and FBS is not expanded by activation, so that each additional IE^+^ cells contributes little to final yields. Alternatively, activation may lower the ability of each IE^+^ cells to produce particles. In either case, infection progress appears to be restricted in most activated LC populations. These restrictions did not appear to affect progeny release (Figure 3A). Rather, activation with most stimuli led to a sharp decline in the amount of intracellular virus produced by each IE^+^ cell, indicating that one or more steps leading to the production of infectious particles, such as capsid egress from the nucleus and virion assembly in the cytoplasm, may be blocked in these cells (Figure 3B).

Mature LC populations were the most severely affected of all. While containing higher numbers of IE^+^ cells, total yields as well as yields per IE^+^ cell are dramatically reduced. Of the three components of the mLC cocktail, CD40L appeared to exert a positive effect on both IE protein expression and yields, while FBS diminished both, so that the efficiency of progeny production per cell of both treatments were similar to that of iLC (Figure 2). LPS, by contrast, had no effect on the number of infected cells but strongly reduced yields, thus lowering the extent of particle production per cell (Figure 2). Combined, CD40L, and LPS increased the number of IE^+^ cells but not yields, further reducing the efficiency of progeny production (Figure 2). This suggests that the mLC phenotype may arise, in part, from the opposing effects of these two molecules: while CD40L signaling may stimulate transcription or translation of the IE proteins, perhaps by acting on the major immediate early genes promoter, LPS signaling may induce the expression of cellular proteins that inhibit viral progeny production.

Very interestingly, the iLC, FBS, CD40L, LPS, and mLC cocktails appeared to have similar effects on viral progeny production starting from genomes introduced by virions during lytic infection as well as from latent viral DNA (Figure 4). This suggests that the restrictions imposed by each stimulus to viral cycle progression are not strongly affected by the action of the tegument proteins released during entry, and can affect progeny production rates independently of the origin of viral DNA (i.e., whether already present in the nucleus or newly introduced by virions). This is an interesting finding, considering that the number and availability of viral genomes should be higher in directly infected cells. Equally interesting is the fact that iLC cultures can support viral reactivation to a similar extent as mature cells. In keeping with our previous results (Coronel et al., 2015), this suggest that differentiation of latently infected CD34^+^ cells into iLC is sufficient to initiate the process of reactivation without an absolute requirement for activation. This is in agreement with data from Reeves et al. (Reeves and Sinclair, 2013), showing that circulating, immature DC express the UL122/123 transcripts and release low amounts of reactivated virus upon co-culture with human fibroblasts, but are in contrast to other reports showing that LPS treatment of circulating DC (Reeves and Sinclair, 2013) and of monocyte-derived LC (Huang et al., 2012) enhance the frequency of reactivation events. The specific reasons for this discrepancy are unclear, but may be related to the fact that we stimulated iLC with LPS in the presence of GM-CSF, thus potentially triggering different signaling pathways.

The only treatment capable of increasing and/or prolonging the incidence of reactivation above that of iLC was exposure to pro-inflammatory cytokines. Inflammation has been associated with increased CMV reactivation rates in immunocompromised (Fietze et al., 1994; Humar et al., 1999; Reinke et al., 1999) and immunocompetent but critically ill patients (Limaye and Boeckh, 2010; Cook and Trgovcich, 2011), and in naturally or experimentally infected myeloid cells (Stein et al., 1993; Prösch et al., 1995; Hahn et al., 1998; Hargett and Shenk, 2010; Reeves and Compton, 2011; Huang et al., 2012; Reeves and Sinclair, 2013). We believe that these stimulatory effects are due to the up-regulation of UL122/123 genes transcription, as it occurs in other cell types after treatment with TNF-α, IL-1β, IL-6, and PGE2 (Stein et al., 1993; Prösch et al., 1995; Kline et al., 1998; Zhu et al., 2002). To our knowledge, this is the first report showing a stimulatory effect of these cytokines, combined, on CMV reactivation from latency.

## Author contributions

LH conceived and designed the experiments; RC, DJ, and LD performed the experiments; LH analyzed the data; JM provided comments and critiques; LH wrote the paper.

### Conflict of interest statement

The authors declare that the research was conducted in the absence of any commercial or financial relationships that could be construed as a potential conflict of interest.
